# *Bacillus clausii* as adjunctive treatment for acute community-acquired diarrhea among Filipino children: a large-scale, multicenter, open-label study (CODDLE)

**DOI:** 10.1186/s40794-019-0089-5

**Published:** 2019-07-23

**Authors:** Jo-Anne A. de Castro, Mary Jean Villa-Real Guno, Marcos O. Perez

**Affiliations:** 1 Department of Pediatrics, De La Salle Health Sciences Institute (DLSHSI) College of Medicine, Cavite, Philippines; 2 Department of Microbiology and Parasitology, Pamantasang Lungsod ng Maynila College of Medicine (PLM-CM), Manila, Philippines; 3 Ateneo School of Medicine and Public Health (ASMPH), Don Eugenio Lopez Sr. Medical Complex, Pasig, Philippines; 4 Department of Pediatric Gastroenterology, Hepatology and Nutrition, The Medical City (TMC), Pasig, Philippines; 5 Sanofi-Aventis Deutschland GmbH, Industriepark Höchst, Building K607, Frankfurt am Main, 65926 Germany

**Keywords:** Acute diarrhea, Children, *Bacillus clausii*, The Philippines, Viral diarrhea, Antibiotic-associated diarrhea

## Abstract

**Background:**

Diarrhea is among the main causes of pediatric mortality in the Philippines. Probiotics have been shown to be beneficial in the management of acute diarrhea. Accordingly, the aim of this population-based study was to assess the safety and effectiveness of *Bacillus clausii* as an adjunct to standard therapy in Filipino children with acute community-acquired diarrhea of viral origin or associated with antibiotic administration.

**Methods:**

A total of 3178 patients (median age of 2 years) were enrolled in this open-label, multicenter, observational study, and were treated with one to two vials of *Bacillus clausii* in the following bacterial stains: O/C, SIN, N/R, and T (oral suspension of 2 billion spores per 5-mL vial) for 5 to 7 days. Diarrhea duration, number of stools per day, improvement in gastrointestinal symptoms, children’s overall acceptability of *Bacillus clausii* therapy, and safety and tolerability were assessed. Concomitant treatment with oral rehydration solutions (26.6%), zinc (23.9%), and antibiotics prescribed for conditions other than diarrhea (13.6%) was recorded during the study. All other probiotics and antidiarrheals were prohibited.

**Results:**

Therapy with *Bacillus clausii* was well-tolerated, and the adverse event rate was very low (0.09%). All reported adverse events, which included vomiting, erythematous rashes and stool color change, were mild to moderate. In more than half of the per-protocol population (1535/2916; 52.6%), diarrhea was resolved within the first 3 days of treatment with *Bacillus clausii*. There was no significant difference (*p* = 0.297) in mean diarrhea duration between patients with either antibiotic-associated (3.3 ± 1.3 days) or viral diarrhea (3.4 ± 1.3 days). However, children who only received *Bacillus clausii* supplementation without zinc had a significantly shorter diarrhea duration (3.3 ± 1.3 days) compared to zinc-treated children (3.6 ± 1.6 days; *p* < 0.001). *Bacillus clausii* significantly reduced the mean number of stools per day, from 5.2 ± 2.0 stools at baseline to 1.2 ± 0.6 stools at study end (*p* < 0.001). Similarly, the proportion of patients with loose stools decreased from 81.6% at baseline to 9.2% at end of treatment period. Acceptability of *Bacillus clausii* therapy was high.

**Conclusion:**

This study adds knowledge on the good safety profile and on the effectiveness of *Bacillus clausii* as an adjunct treatment for acute childhood diarrhea.

## Background

In 2015, diarrheal disease caused more than 650 million illnesses and approximately 500 000 deaths worldwide among children under five years of age [1]. Diarrhea is also among the main causes of pediatric mortality in the Philippines, responsible for the death of 10 000 Filipino children every year [2].

In the management of acute diarrhea, treatment is largely supportive. Oral rehydration therapy (ORT) continues to be the cornerstone of treatment, whereas the use of antibiotics is discouraged [3]. Many years ago, probiotics were introduced as adjuvant therapy for the management of diarrhea [4]. Probiotics have been shown to protect the enteric microflora through the production of anti-pathogenic compounds [5–7]. They also exhibit immunomodulatory activities, and regulate immune signaling pathways [5, 8, 9]. In order to treat and prevent diarrhea, probiotics work to preserve the integrity of the intestinal mucosa and enhance the equilibrium of electrolytes [10]. Probiotics currently in clinical use include bacteria which are normal inhabitants of the human gastrointestinal (GI) tract such as lactic acid bacteria, and spore-forming bacteria which are not found in the GI tract, mainly members of the genus *Bacillus* [11].

*Bacillus clausii* has been on the market for over 55 years, and is characterized by the presence of four probiotic strains (O/C, SIN, N/R and T) [5]. Its resistance to both physical and chemical conditions (such as heat, antibiotics, and gastric pH) is attributed to its spores [12, 13]. As *Bacillus clausii* is highly resistant to most antibiotics, its efficacy is not altered by concomitant antibiotherapy [13]. Moreover, *Bacillus clausii* is unique by its fast growth in aerobic and anaerobic environments [14].

The efficacy of *Bacillus clausii*, as adjunct therapy to ORT with or without zinc supplementation in children with acute diarrhea, was documented in several randomized controlled clinical trials [15–18]. Furthermore, *Bacillus clausii* was found to reduce the incidence and intensity of the most common side effects of anti-*Helicobacter pylori* antibiotherapy (e.g., antibiotic-associated diarrhea [AAD]) compared to placebo, in symptom-free, *Helicobacter pylori*-positive adults [19]. In the Philippines, *Bacillus clausii* was granted marketing authorization in 2005. The aim of this large-scale, multicenter, observational, population-based study was to assess the safety, acceptability, and effectiveness of *Bacillus clausii* as an adjunct to ORT and/or zinc supplementation in Filipino children with acute community-acquired diarrhea of viral origin or associated with antibiotic administration (Study Acronym: CODDLE).

## Methods

*Bacillus* ***c****lausii* in the treatment **o**f acute community-acquire**d d**iarrhea among Fi**l**ipino childr**e**n (CODDLE) was a prospective, open-label, multicenter, observational study conducted between January 2007 and October 2010. Participating investigators were pediatricians and general practitioners. The study was conducted in accordance with the Declaration of Helsinki, and the International Council for Harmonization − Good Clinical Practice guidelines. Informed consent was obtained from all participants prior to their enrollment. The study protocol was approved by the research ethics committees at each study site.

### Patients

Children between 1 month and 6 years of age with community-acquired diarrhea lasting for less than 48 h, which was either antibiotic-associated or viral, were included. Patients had to have three or more watery stools during the preceding 24 h. Patients were excluded if they had bloody stools, hospital-acquired diarrhea, or infectious diarrhea requiring antibiotic therapy. Moreover, patients with chronic diarrhea of any origin (e.g., Crohn’s disease, hemorrhagic rectocolitis, and irritable bowel syndrome), intractable vomiting, abdominal distension, severe dehydration, or a history of seizures were also excluded from the study. Finally, patients were excluded if they had any condition known to produce immunodeficiency or co-morbidities such as pneumonia, typhoid and urinary tract infection, or if they were allergic to *Bacillus clausii*, or receiving other probiotics.

### Study treatments

Eligible subjects were treated with one to two vials of *Bacillus clausii* (Erceflora®, *Sanofi*, Philippines) per day for 5 to 7 days depending on the age of the child and the severity of the diarrhea, with each 5-mL vial containing an aqueous suspension for oral administration of 2 billion spores of *Bacillus clausii* in the following four bacterial stains: O/C, SIN, N/R, and T.

Concomitant treatment with antacids and adsorbents such as bismuth subsalicylate, enkephalinase inhibitors such as acetorphan, oral rehydration solutions, zinc supplements, and antibiotics prescribed for conditions other than diarrhea was permitted and was recorded during the study. Similarly, all nutritional preparations containing prebiotics and probiotics (e.g., milk formula) were also permitted and documented, whereas all other probiotics and antidiarrheal drugs were prohibited during the study, unless deemed necessary by the investigator.

### Study assessments

Subjects were evaluated at baseline and 7 days after the start of the study medication. At baseline, the patient information routinely collected for this study included patient demographics, medical history, and concomitant medications. A daily diary was also handed out to the parents/legal guardians to monitor the number of *Bacillus clausii* vials taken, the number and characteristics (loose, watery, lumpy, or formed) of stools per day according to a photo scale, concomitant medications, adverse events (AEs), and the nutritional preparations containing prebiotics and probiotics taken during the study, if any. Parents/legal guardians were thoroughly trained in how to use the diary, and investigators evaluated the parents/legal guardians’ ability to fill out the stool diary correctly. Patients were instructed to come back for follow-up 7 days after the start of the study medication with the daily diary, for a final evaluation.

Safety evaluation included the frequency and severity of AEs, as well as their relationship with the study drug; serious AEs; discontinuation due to AEs; physical examination; and vital signs. Effectiveness measures included the duration of diarrhea, as counted from the first intake of the study drug up to the first appearance of a loose stool followed by two consecutive normal stools; the mean number of stools per day; and the improvement in GI symptoms, such as nausea, vomiting and abdominal pain. Children’s overall acceptability of *Bacillus clausii* therapy, as judged by the parents/legal guardians using a 4-point Likert’s scale, was also evaluated.

### Statistical considerations

The intention-to-treat (ITT) population included all patients who received at least one dose of *Bacillus clausii*, and was used to evaluate the safety profile of *Bacillus clausii* in this study. The per-protocol (PP) population included all patients who took at least one dose of *Bacillus clausii* and had at least one post-baseline effectiveness measurement.

Sample size calculation was not based on specific statistical tests and formulas, as one of the aims of this study was to fulfill the requirements of the Philippines Food and Drug Administration (previously known as the Bureau of Food and Drugs), in terms of reporting of the therapeutic effects and adverse reactions of newly introduced products in 1000 patients per year or 3000 patients over 3 years.

Continuous variables were presented as numbers, means, medians, and standard deviations (SD). Counts and percentages were used for categorical variables. AEs were reported in counts and percentages, and were summarized by severity and relation to study treatment. Chi-square test of independence, Wilcoxon two-sample test, repeated measures analysis of variance (ANOVA), and McNemar’s test were used for data analysis and to evaluate differences between patient subgroups.

The safety and effectiveness outcomes were evaluated in the entire study population, and based on the cause of diarrhea (viral or antibiotic-associated). In all statistical analyses, *p* < 0.05 (two-sided test) was considered significant.

## Results

### Patients’ characteristics

**Fig. 1:**
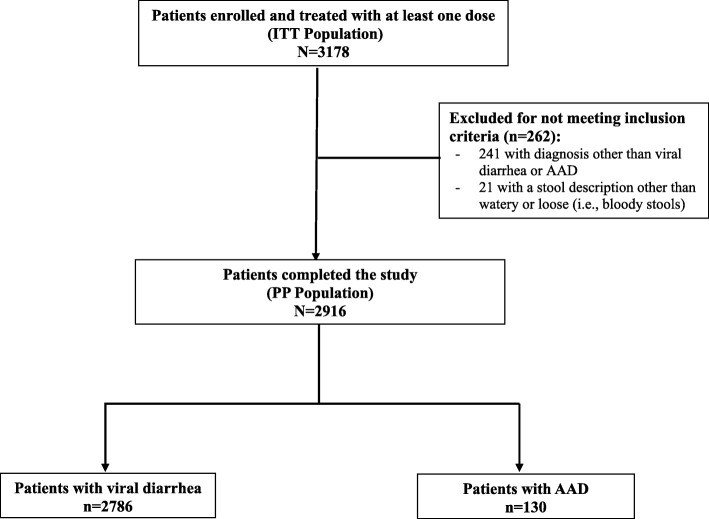
Flowchart of study population. AAD, antibiotic-associated diarrhea; ITT, intention-to-treat; PP, per-protocol

A total of 3178 patients, with a median (interquartile range) age of 2 (1 to 4) years, were enrolled and formed the ITT population. Out of these patients, 2916 (91.8%) formed the PP population that excluded 262 subjects for not meeting the study’s inclusion/exclusion criteria (Fig. 1). In the PP population, 2786 patients (95.5%) were diagnosed with community-acquired diarrhea of viral origin and 130 (4.5%) with AAD.

Patient demographics and baseline characteristics of the ITT population are presented in Table 1. The male to female ratio was 1.2:1, and there was no gender difference across the age groups. The PP population presented similar demographic and baseline characteristics as the ITT population. At study entry, the most common signs of dehydration in the ITT population were thirst in 1594 patients (50.2%) and dry skin in 930 patients (29.3%). Moreover, most patients presented at baseline GI symptoms, such as vomiting (in 43.7% of the ITT population), abdominal pain (25.2%), nausea (15.6%), and abdominal bloating (12.4%).

**Table 1:** Patient demographics and baseline characteristics of the intention-to-treat (ITT) population

	ITT population (*N* = 3178)
Age (years), median [interquartile range]	2.0 [1.0–4.0]
Gender	
Female, n (%)	1432 (45.1)
Male, n (%)	1746 (54.9)
Weight (kg)	15.1 (10.3)
Height (cm)	88.3 (28.1)
Pulse (beats per minute)	98.7 (18.8)
Respiratory rate (breaths per minute)	29.4 (10.2)
Body temperature (°C)	37.4 (10.6)

Data are expressed as mean (standard deviation), unless otherwise specified

During the study, less than 1% (30/3178) of patients received nutritional preparations containing prebiotics or probiotics, and 50.8% (1613/3178) of patients received other concomitant medications. These included antibiotics for conditions other than diarrhea (*n* = 431; 13.6%), zinc supplements (*n* = 759; 23.9%), and ORT (*n* = 846; 26.6%). Table 2 illustrates the use of concomitant medications in the PP population (*N* = 2916).

**Table 2:** Concomitant medication use in the per-protocol (PP) population

	Viral diarrhea *N* = 2786 n (%)	AAD *N* = 130 n (%)	Total *N* = 2916 n (%)
At least one concomitant medication	1389 (49.9)	107 (82.3)	1496 (51.3)
ORT	778 (27.9)	40 (30.8)	818 (28.1)
Zinc supplementation	715 (25.7)	33 (25.4)	748 (25.7)
Antibiotics for conditions other than diarrhea	303 (10.9)	66 (50.8)	369 (12.7)
Other concomitant medications	299 (10.7)	34 (26.2)	333 (11.4)
Nutritional preparations containing prebiotics or probiotics	24 (0.86)	2 (1.5)	26 (0.89)

Percentages are calculated as n/N

*AAD*, antibiotic-associated diarrhea; *ORT*, oral rehydration therapy

### Safety and tolerability

The mean ± SD duration of treatment with *Bacillus clausii* in the ITT population (*N* = 3178) was 5.8 ± 1.3 days, with a median total dose of 10 vials or 2 vials per day.

A total of 3 AEs were reported by 3/3178 patients (0.09%) during the study. These included vomiting for which the relationship to the study medication could not be determined, erythematous rashes deemed unrelated to *Bacillus clausii*, and stool color change (green stool) which was judged as being possibly related to the study medication. All AEs were mild to moderate in severity, and all the patients recovered from the events without sequelae at the end of the study. Vomiting was the only AE that led to discontinuation of *Bacillus clausii* therapy.

No serious AEs or deaths were reported, and no clinically relevant changes in vital signs were observed over the course of treatment.

### Overall effectiveness outcomes

**Fig. 2:**
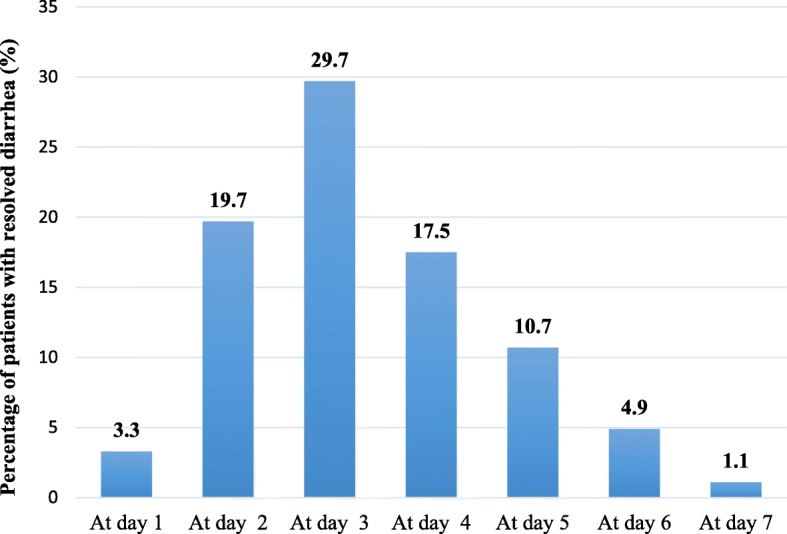
Percentage of patients with resolved diarrhea during the study in the per-protocol population (*N* = 2916)

In the PP population, 2534/2916 patients (86.9%) had resolved diarrhea during the 7-day treatment period with *Bacillus clausii* vials. The median duration of diarrhea was 3 days, and there was no statistically significant difference (*p* = 0.297; Wilcoxon two-sample test) in the mean duration of diarrhea between patients with AAD (3.3 ± 1.3 days) and patients with viral diarrhea (3.4 ± 1.3 days). Similarly, there was no statistically significant difference (*p* = 0.742) in mean diarrhea duration between children who received nutritional preparations containing prebiotics or probiotics (3.2 ± 0.9 days) and those who did not (3.4 ± 1.3 days). However, children who did not receive zinc supplementation had a significantly shorter mean diarrhea duration (3.3 ± 1.3 days) compared to those who did (3.6 ± 1.6 days; *p* < 0.001). Furthermore, although *p*-values were not calculated for this parameter, it was found that the influence of zinc supplementation on the mean duration of diarrhea depended on the isolated organism. In the AAD subgroup, the mean duration of diarrhea was 3.2 ± 1.4 in children treated with zinc versus 3.3 ± 1.3 days in those without zinc supplementation. In contrast, in the viral diarrhea subgroup, mean diarrhea duration was 3.6 ± 1.4 in zinc-treated children and 3.3 ± 1.3 days in those without zinc supplementation. In more than half of the PP population (1535/2916; 52.6%), diarrhea was resolved within the first 3 days of treatment with *Bacillus clausii* (Fig. 2).

**Fig. 3:**
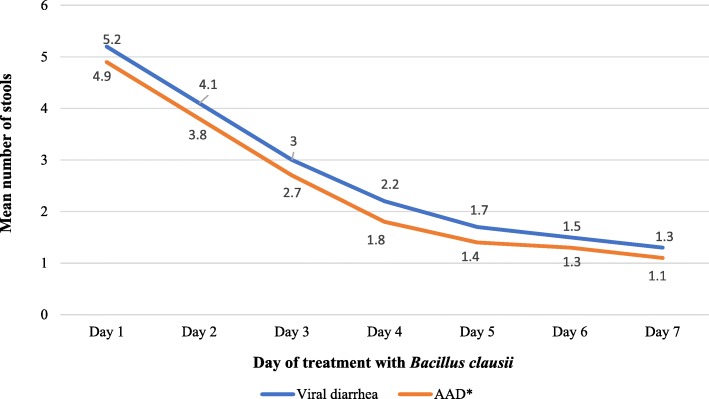
Mean number of stools per day of treatment, according to the cause of diarrhea. *AAD, antibiotic-associated diarrhea. Repeated measures analysis of variance: viral diarrhea versus AAD: *p* = 0.938

The number of diarrheal episodes significantly decreased (*p* < 0.001) at each day of treatment, from a median of 5 episodes per day at baseline to 1 episode per day starting from the fifth day of treatment. Moreover, administration of *Bacillus clausii* significantly reduced the mean number of stools per day in the PP population, from 5.2 ± 2.0 stools at baseline to 1.3 ± 0.6 stools after 7 days of treatment (*p* < 0.001). The decrease in the mean number of daily stools was not significantly different (*p* = 0.938; repeated measures ANOVA) between patients with viral diarrhea (5.2 ± 2.0 stools at baseline versus 1.3 ± 0.6 stools after 7 days of medication start) and those with AAD (4.9 ± 1.9 stools at baseline versus 1.1 ± 0.4 stools after 7 days) (Fig. 3). Furthermore, the proportion of patients in the PP population with loose stools decreased from 81.6% (2379/2916) at baseline to 9.2% (268/2916) at the end of the treatment period (*p* < 0.001).

**Fig. 4:**
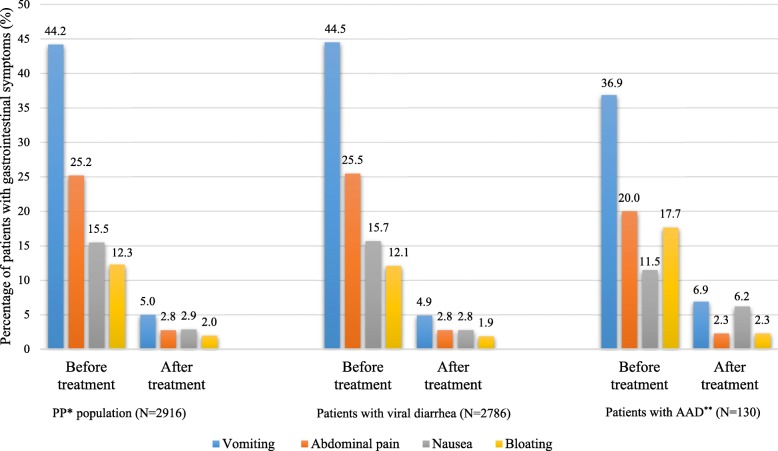
Percentage of patients experiencing gastrointestinal symptoms before and after *Bacillus clausii* treatment. *PP, per-protocol; **AAD, antibiotic-associated diarrhea. Before treatment versus after treatment (in the overall PP population): *p* < 0.001 (McNemar’s test). Before treatment versus after treatment (in the viral diarrhea subgroup): *p* < 0.001 (McNemar’s test). Before treatment versus after treatment (in the AAD subgroup): *p* < 0.001 (except for nausea: *p* = 0.083) (Chi-square test). Viral diarrhea versus AAD: *p* > 0.05 (Chi-square test)

The incidence rate of all four GI symptoms (nausea, vomiting, abdominal pain, and bloating) significantly decreased from baseline to study end, in the overall PP population and in both subgroups (*p* < 0.001 for both analyses), with the exception of nausea in the AAD subgroup for which the incidence was only moderately decreased throughout the study (*p* = 0.083) (Fig. 4).

### Parent/legal guardians’ assessment

According to the parents/legal guardians’ assessments, children’s overall acceptability of *Bacillus clausii* therapy was considered “good” to “excellent” in 97.6% (3101/3178) of the ITT population.

## Discussion

To our knowledge, CODDLE is the first large-scale study in the Philippines investigating the real-world safety, acceptability, and effectiveness of *Bacillus clausii* in the treatment of children with acute community-acquired diarrhea.

*Bacillus clausii* is an aerobic, spore-forming bacterium that is able to inhibit the growth of pathogens in the GI tract [20]. Several mechanisms have been proposed to explain the effect of *Bacillus clausii* against acute childhood diarrhea. The mechanisms by which spore-forming probiotics could reduce acute diarrhea include: stimulation of the immune system, synthesis of different antimicrobial substances, such as bacteriocins and enzymes, and modulation of the composition of gut microbiota [21]. The potential mechanism behind establishment of gut homeostasis involves promotion of growth of other beneficial microbes, and suppression of pathogen and pathogen-induced inflammatory response of intestinal mucosa [21].

Despite these beneficial properties of *Bacillus clausii*, empirical evidence for the clinical effectiveness of probiotics has shown mixed results, and very little is still known about which probiotics work for which indication and group of patients [22]. However, in recent years, the use of probiotics for the treatment of acute childhood diarrhea has increasingly become an area of research interest [22, 23]. Diarrhea in young children can have severe consequences, hence finding safe and effective interventions to reduce the duration or the severity of symptoms is pertinent [22].

Probiotics are recognized for their long history of safe use [21]. In this study, therapy with *Bacillus clausii* was well-tolerated, and the rate of reported AEs was very low. This finding is consistent with the safety results of several randomized controlled studies, in which *Bacillus clausii* demonstrated a good safety profile [16, 18, 24]. In addition, the favorable safety profile observed in CODDLE is in line with the results of a recent systematic review and meta-analysis of six randomized controlled trials, investigating the effects of *Bacillus clausii* in acute childhood diarrhea, which did not report any serious AEs related to *Bacillus clausii* [25]. Moreover, the safety of *Bacillus clausii* was supported by a 2004 double-blind, randomized, placebo-controlled trial, which assessed the effects of *Bacillus clausii* vials in 120 adult patients with AAD due to anti-*Helicobacter pylori* therapy. It was found that the individual patients’ overall assessment of tolerability was significantly better in the group treated with *Bacillus clausii* than in the placebo group (*p* < 0.05 after 2 weeks of treatment) [19]. Indeed, the CODDLE study did not identify new safety issues or concerns compared to the known safety profile of the product; vomiting which was experienced by one study participant was reported to be a common AE of *Bacillus clausii* supplementation in the meta-analysis evaluating *Bacillus clausii* in acute childhood diarrhea [25]. Hypersensitivity reactions, including skin rashes which was reported by one patient in our study, were listed as possible AEs of *Bacillus clausii* supplementation in the product’s prescribing information [26].

Our study indicates a reduction in diarrhea duration, incidence of GI symptoms, and stool frequency associated with *Bacillus clausii* administration, which may be indicative of attenuated intensity of the intestinal infection [27]. These results are in line with the findings of the 2018 meta-analysis of randomized controlled trials of *Bacillus clausii* in the treatment of acute childhood diarrhea. Data arising from pooled analysis showed that *Bacillus clausii* therapy combined with ORT significantly reduced the duration of diarrhea (mean difference of − 9.12 h; *p* = 0.015) and the duration of hospitalization (mean difference of − 0.85 days; *p* = 0.017), compared to ORT with or without zinc supplementation. There was also a trend of decreasing stool frequency after *Bacillus clausii* administration combined with ORT, compared to controls (mean difference of − 0.19 diarrheal motions). Thus, according to this systematic review, *Bacillus clausii* might represent an effective therapeutic option in acute childhood diarrhea [25].

The results of our study regarding the effectiveness of *Bacillus clausii* in acute childhood diarrhea of viral origin or associated with antibiotic administration are also in line with findings from randomized controlled trials investigating the effect of *Bacillus clausii* in adults with AAD. The study of Nista et al. (2004) found a greater reduction in the risk of diarrhea in the *Bacillus clausii* group compared with the placebo group after one (relative risk [RR] of 0.30; *p* < 0.01) and two weeks (RR = 0.38) of treatment, as well as significantly lower incidences of epigastric pain and nausea (*p* < 0.05 for both events) [19]. In addition, a meta-analysis on probiotics (*Lactobacillus*, *Bifidobacterium*, *Saccharomyces, Streptococcus*, *Enterococcus*, and/or *Bacillus*) for the prevention and treatment of AAD showed encouraging results [28]. The pooled RR across 63 randomized controlled trials indicated a significant association of probiotic administration with reduction in AAD (RR = 0.58; 95% confidence interval, 0.50 to 0.68; *p* < 0.001). However, this meta-analysis was limited by poor documentation of the probiotic strains, and lack of assessment of probiotic-specific AEs [28].

Despite the 2004 recommendations of the World Health Organization to treat all types of diarrhea among all age groups with zinc tablets in association with ORT [29], we found that the influence of zinc supplementation on the mean duration of diarrhea depended on the isolated organism. The results of our study showed that zinc supplementation reduced mean diarrhea duration in children with AAD, yet it had a detrimental effect in children with viral diarrhea. These findings are in line with a 2010 analysis of a double-blind, randomized, placebo-controlled trial conducted in 801 children with acute diarrhea aged 6 to 59 months in India [30]. This study showed that zinc supplementation had a strikingly protective effect when *Klebsiella* was isolated, no effect when single *Escherichia coli* or intestinal parasites were isolated, but a high risk of prolonged diarrhea when rotavirus was isolated and an even higher likelihood (3.4 times) of prolonged diarrhea when *Escherichia coli* was isolated along with rotavirus [30]. Similarly, in a previous double-blind, randomized, placebo-controlled study which aimed to determine the efficacy of zinc in reducing stool output and diarrheal duration when administered as an adjunct to ORT in 287 hospitalized children in India aged 3 to 36 months with acute diarrhea and dehydration, the beneficial effect of zinc could not be observed in rotavirus-infected children [31]. Thus, it is clear that the beneficial effect of zinc supplementation in acute diarrhea is not equal against all organisms isolated from stools [29, 30]. Hence, our findings, along with previous research results [30, 31], suggest that in children with acute diarrhea of viral origin, zinc should be used cautiously.

In our study, children’s overall acceptability of *Bacillus clausii* therapy was rated as “good” to “excellent” by the vast majority (> 95%) of the parents/legal guardians. This high level of acceptability reported in this study might be partly explained by the tasteless and odorless properties of the study medication.

The present study had some limitations. Since this was an observational study, a common and predesigned treatment strategy was absent. Thus, the findings reported here should be interpreted within the context of a clinical observation. Moreover, an analysis by study center was not performed, and the impact of any between-center differences on the study results was not taken into account. Furthermore, the majority of the data collected in this study depended on what the parents/legal guardians reported in their daily diary, hence exposing the study to a measurement bias. However, assessing outcomes reported by the parents/legal guardians could be useful for physicians’ communication with parents about the management of children who have diarrhea [32]. Finally, the effectiveness results of the CODDLE study are based on the PP analysis, as an ITT analysis was not used to assess the effectiveness of *Bacillus clausii* as adjunctive therapy in acute childhood diarrhea. Despite these limitations, being multicenter with a large sample size, this study stands as a reliable source for the use of *Bacillus clausii* in daily clinical practice. Moreover, since probiotics are likely to have different effects depending on the nature of the causative agent, this study evaluated the effectiveness of *Bacillus clausii* while taking into consideration the etiology of diarrhea.

## Conclusions

CODDLE, a population-based observational study, documented the good safety and tolerability profile of *Bacillus clausii* in the management of acute childhood community-acquired diarrhea of viral origin or associated with antibiotic administration. Furthermore, this study adds knowledge on the effectiveness of *Bacillus clausii* encompassing the four probiotic strains, namely O/C, SIN, N/R, and T, as an adjunct treatment for children with acute diarrhea. In addition, our results add to the growing body of evidence that the beneficial effect of zinc may not be equivalent against the causative agents of acute diarrhea. This study indicated that the combination of *Bacillus clausii* with zinc has a positive impact in children with AAD, but that zinc should be used cautiously in children with viral diarrhea.

## Data Availability

All data generated or analyzed during this study are included in this published article.

## Abbreviations

AAD: Antibiotic-Associated Diarrhea
AE: Adverse Event
ANOVA: Analysis of Variance
GI: Gastrointestinal
ITT: Intention-to-Treat
ORT: Oral Rehydration Therapy
PP: Per-Protocol
RR: Relative Risk
SD: Standard Deviation
